# CD47 in the Brain and Neurodegeneration: An Update on the Role in Neuroinflammatory Pathways

**DOI:** 10.3390/molecules26133943

**Published:** 2021-06-28

**Authors:** Seyed Mohammad Gheibihayat, Ricardo Cabezas, Nikita G. Nikiforov, Tannaz Jamialahmadi, Thomas P. Johnston, Amirhossein Sahebkar

**Affiliations:** 1 Department of Genetics, School of Medicine, Shahid Sadoughi University of Medical Sciences, Yazd 8916188635, Iran; gheibi65@yahoo.com; 2 Department of Physiology, School of Medicine, Universidad Antonio Nariño, Bogotá 111511, Colombia; ricardocabe@gmail.com; 3 Laboratory of Cellular and Molecular Pathology of Cardiovascular System, Institute of Human Morphology, Tsyurupa Street, 117418 Moscow, Russia; nikiforov.mipt@googlemail.com; 4 Laboratory of Medical Genetics, Institute of Experimental Cardiology, National Medical Research Center of Cardiology, 121552 Moscow, Russia; 5 Department of Food Science and Technology, Quchan Branch, Islamic Azad University, Quchan 19395/1495, Iran; jamiat931@gmail.com; 6 Department of Nutrition, Faculty of Medicine, Mashhad University of Medical Sciences, Mashhad 13944-91388, Iran; 7 Division of Pharmacology and Pharmaceutical Sciences, School of Pharmacy, University of Missouri-Kansas City, Kansas City, MO 64131, USA; johnstont@umkc.edu; 8 Biotechnology Research Center, Pharmaceutical Technology Institute, Mashhad University of Medical Sciences, Mashhad 13944-91388, Iran; 9 Applied Biomedical Research Center, Mashhad University of Medical Sciences, Mashhad 13944-91388, Iran; 10 School of Medicine, The University of Western Australia, Perth 6907, Australia; 11 School of Pharmacy, Mashhad University of Medical Sciences, Mashhad 13944-91388, Iran

**Keywords:** CD47, SIRPα, neurodegeneration, multiple sclerosis, stroke, Alzheimer, Parkinson

## Abstract

CD47 is a receptor belonging to the immunoglobulin (Ig) superfamily and broadly expressed on cell membranes. Through interactions with ligands such as SIRPα, TSP-1, integrins, and SH2-domain bearing protein tyrosine phosphatase substrate-1 (SHPS-1), CD47 regulates numerous functions like cell adhesion, proliferation, apoptosis, migration, homeostasis, and the immune system. In this aspect, previous research has shown that CD47 modulates phagocytosis via macrophages, the transmigration of neutrophils, and the activation of T-cells, dendritic cells, and B-cells. Moreover, several studies have reported the increased expression of the CD47 receptor in a variety of diseases, including acute lymphoblastic leukemia (ALL), chronic myeloid leukemia, non-Hodgkin’s lymphoma (NHL), multiple myeloma (MM), bladder cancer, acute myeloid leukemia (AML), Gaucher disease, Multiple Sclerosis and stroke among others. The ubiquitous expression of the CD47 cell receptor on most resident cells of the CNS has previously been established through different methodologies. However, there is little information concerning its precise functions in the development of different neurodegenerative pathologies in the CNS. Consequently, further research pertaining to the specific functions and roles of CD47 and SIRP is required prior to its exploitation as a druggable approach for the targeting of various neurodegenerative diseases that affect the human population. The present review attempts to summarize the role of both CD47 and SIRP and their therapeutic potential in neurodegenerative disorders.

## 1. Introduction

CD47, known as cluster of differentiation 47 or IAP (integrin associated protein), is a widely expressed transmembrane glycoprotein of 50 kDa belonging to the immunoglobulin (Ig) superfamily, which possesses 5 transmembrane domains of interaction. Through interactions with its ligands such as SIRPα, TSP-1, integrins, and SHPS-1, CD47 regulates numerous functions like cell adhesion, proliferation, apoptosis, migration, homeostasis, phagocytosis via macrophages–“don’t eat me signal”, neutrophils migration, and T-cells, B-cells and dendritic cells activation [1,2]. Moreover, several studies have shown that CD47 receptor expression is significantly increased in a variety of diseases, including non-Hodgkin’s lymphoma (NHL), acute myeloid leukemia (AML), acute lymphoblastic leukemia (ALL), chronic myeloid leukemia, multiple myeloma, bladder cancer, Gaucher disease, Multiple Sclerosis and stroke among others [3,4,5].

The expression of a signal regulatory protein (SIRP), also known as CD172 and its ligand CD47 (also known as integrin-related protein), has been demonstrated to occur on the surface of microglial cells, hippocampal neurons, olygodendrocytes, and astrocytes. In this aspect, it seems like the interaction of SIRP with CD47 is crucial for cell-to-cell communication in the brain both in normal and pathological conditions [5,6,7]. During neurological disorders, CD47 functions as a key neuroimmune modulator responding to chronic and acute CNS injuries and in the process of recovery [5,8]. Additionally, CD47 has broad involvement in the death of neuronal cells, inflammation, and the development of acute ischemic brain injuries [9,10]. The present review attempts to summarize the role of CD47 and its associated receptor SIRPα (signal regulatory protein α) during the development of neurodegenerative disorders such as Multiple Sclerosis, Stroke, Parkinson’s, Alzheimer and traumatic brain injury among others. Finally, the therapeutic potential of SIRP and CD47 as reported by different models will also be examined.

## 2. Neuroimmune Regulatory Proteins (NIRegs) and CD47 in the CNS

**Figure 1:**
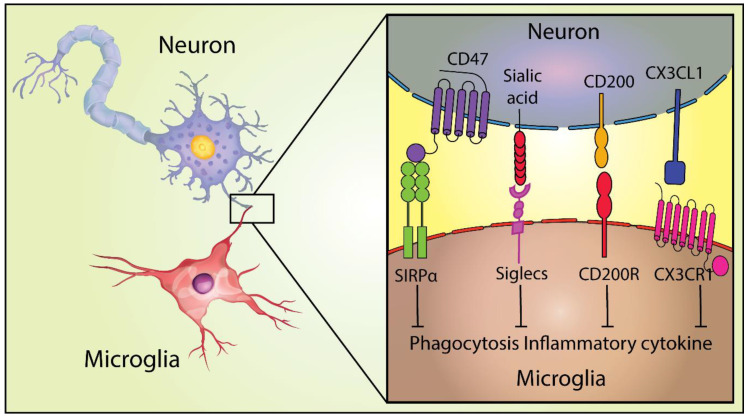
NIREGs such as CD47 provide “don’t eat me signals” and keep microglia in a homeostatic state. Adapted from [14].

NIREGs are functionally neuroimmune proteins involved in the regulation of the innate immune response and the duration of inflammatory response in the host (Figure 1). These regulators include the proteins CX3CL1, CD47, and CD200, constitutively expressed by neurons, astrocytes, oligodendrocytes, and microglia, and involved in tissue resilience [11,12]. Moreover, CD47, CD95L, CD55, CD200, fH, and CD46, all NIREGs, have been shown to contribute to downregulate immunity at the molecular and cellular levels and, thereby, suppress inflammation in the brain and other organs [13].

Interestingly, it has been shown that oligodendrocytes can express both CD47 and CD200 in order to control microglia behavior and inhibit specific immune reactions in the CNS [15]. Furthermore, oligodendrocytes express SEMA3A [16], which plays a role in inducing microglia apoptosis [17]. Additionally, the expression of NIRegs via oligodendrocytes is highly important in the modulation of the immune response in the brain [18]. Furthermore, another study determined that oligodendrocytes also express FasL which is a type-II transmembrane protein that belongs to the tumor necrosis factor (TNF) family [19]. FasL is able to potentially stimulate apoptosis on FAS (*F*S-7-associated surface antigen) expressing activated T and NK cells [20] and as a result, limits the inflammatory processes in the brain [15].

Other NIREGS such as CD47, CD200, and CX3CL1 are important in tissue resilience in neurons or through the interaction with co-receptors (SIRPα, CD200R, and CX3CR1) in microglia, modulating the phenotypes from resting to an activated state, among other functions [14]. Importantly, it was recently shown that the expression patterns of both CD47 and SIRPα, prevent excessive microglial phagocytosis and synaptic pruning in the dorsal lateral geniculate nucleus in mice [21]. In the following sections, the numerous roles of CD47 and its receptor SIRPα will be explored along with their signaling mechanisms.

### CD47 Signaling Interactions: The Role of SIRPα

SIRPα is an inhibitory receptor with high-affinity interaction for CD47 in brain cells and other tissues. This protein belongs to the SIRP family of receptors which comprise SIRPα, SIRPβ, and SIRPγ as well as soluble SIRPδ members [5,22]. Accumulating evidence shows that the interaction between SIRP and CD47 is important in mediating the cell-cell communication in the brain through different modulatory processes of the microglia [5]. In this aspect, both SIRPα and SIRPβ1 have been shown to be expressed in microglia with different homeostatic functions [23].

Furthermore, various studies have examined the structural interactions and signaling pathways activated by SIRPα/CD47 [24]. In this aspect, both structural and mutagenesis research have produced information concerning structural requirements for the interaction between SIRPα and CD47. Extracellular portions of SIRPα consist of three IgSF domains, including two membrane-proximal IgC domains and one membrane-distal IgV domain (N-terminal). Previous research has shown that the N-terminal IgV domain of SIRPα binds to CD47 [25]. However, for CD47, the extracellular IgV-like fold is responsible for its interactions with SIRPα and its association with three integrins [23]. Within the SIRPα/CD47 complex, four loop structures (i.e., the BC, C’D, DE, and FG loops) at the end of the IgV SIRPα domain create an interface for the specific structural features of CD47 (i.e., the BC and FG loops, C’ strand around the FG loop, and N-terminal pyroglutamic acid) [24]. Though extracellular areas of SIRPα and SIRPβ1 have a degree of homology, CD47 binds to SIRPα (and SIRPγ), but it does not bind to SIRPβ1 [23]. Results from other studies have ascribed the aforementioned binding specificity to supplemental loops within SIRPs, which allow minor sequence modifications that can produce significant alterations in binding affinity [24]. In addition, SIRPβ1 expression has been determined to occur on the cell surface as a disulfide-linked homodimer, which forms via Cys-320-mediated binding in the membrane-proximal Ig loop [26]. However, currently, there is insufficient information about the SIRPβ ligand.

**Figure 2:**
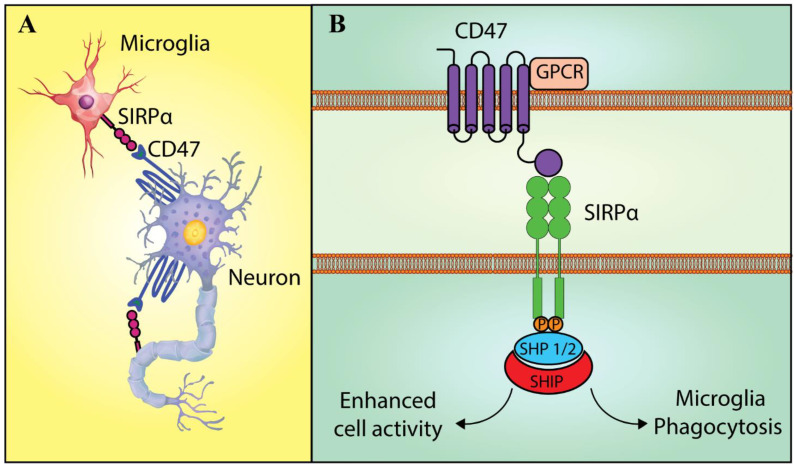
The cell-to-cell cross-talk via the SIRPα-CD47 signaling. (**A**) Bi-directional signaling between CD47 and SIRPα. CD47 and SIRPα are possibly co-expressed on a similar cell and their ligation could mediate the inter-cellular signaling in a bi-directional manner. Even though SIRPα-CD47 signaling in the microglia is incompletely understood, the specific contribution of SIRPα and CD47 has been examined. Moreover, CD47 and SIRPα interactions could be observed in the phagocytic function of the microglia. (**B**) SIRP signaling in microglia. SIRPα phosphorylation enables the docking and the recruitment of SHP-1 and SHP-2. In this regard, various studies have shown that SHP-1 and SHP-2 perform opposite biological functions. Different signaling pathways have been negatively regulated by SHP-1 for the inhibition of numerous cell functions like phagocytosis. On the contrary, events affecting the cellular activity such as migration, as well as growth, have been shown to be positively regulated by SHP-2 [5]. Abbreviation: GPCR: G protein-coupled receptor, SIRPα: Signal regulatory protein α, SHP 1/2: SH2 domain-containing phosphatases 1 and 2, SHIP: SH2 domain-containing inositol phosphatase.

Basically, receptors in the immunoglobulin superfamily react with the ‘turning off’ signal and thus modulate microglia activation, migration, and phagocytosis [27]. This regulation occurs through a cytoplasmic-domain, immuno-receptor tyrosine-based inhibition motif (ITIM), whereas receptor activation during microglia regulation occurs by a cytoplasmic-domain ITAM [27]. It is notable that the cytoplasmic region of SIRPα, as one of the receptors that recognize the off-signal, consists of two ITIM with four strongly conserved tyrosine residues. In addition, SIRPα ligation via CD47 would induce phosphorylation of these tyrosine residues. Moreover, both C-terminal tyrosine phosphorylation sites; that is, the Y^47^ and the Y^449^ sites, create the docking sites for the inhibitory phosphatases SH2 domain-containing phosphatase (SHP)-1 and SHP-2 [28]. Hence, these phosphorylated residues work as de-phosphorylation sites for SHPs. Notably, SHP-2 and SHP-1 exert opposite biological functions [29] (Figure 2). According to some studies, SHP-1 negatively regulates diverse signaling paths to inhibit numerous cell functions. In contrast, SHP-2 positively regulates the signaling events that contribute to cellular activity like migration and growth. As an example, research has demonstrated that SIRPα is involved in the inhibition of macrophage phagocytosis by recruiting SHP-1 [30,31].

## 3. Bi-Directional Signaling between CD47 and SIRPα

One aspect that is specific to SIRPα-CD47 signaling is that both receptors may be co-expressed on a similar cell, and their subsequent ligation could potentially mediate intercellular signaling in a bi-directional manner [23] (Figure 2). The above property would be of particular importance in the CNS, wherein large overlaps in CD47 and SIRPα expression have been observed [30]. As an example, the expression of CD47 and SIRPα have been observed on the surface of hippocampal neurons; that is, SIRPα is found on dendrites and axons, whereas CD47 is limited to dendrites [30]. Another study showed the significant contribution of CD47-SIRPα interactions between neighboring neurons to the formation of the neuronal network in the hippocampus [32]. Accordingly, co-localization of CD47 and SIRPα has been observed in microglia, whereas myelin, Schwann cells, oligodendrocytes, and astrocytes express CD47 without SIRPα [33].

Even though SIRPα-CD47 signaling in microglia is not completely understood, the specific involvement of SIRPα with CD47 continues to be intensively investigated. As previously mentioned, both SIRPα and CD47 play a role in the phagocytic function of microglia, and in this aspect, the inhibition of microglial phagocytosis is mediated by SIRPα. Also, it has been reported that CD47 cooperates with other surface receptors like TLR4, CD36, scavenger receptor A, TLR2, and integrinα_6_β_1_ as a complex and affects microglial interaction with Aβ fibrils [34]. Subsequently, phagocytosis of Aβ fibrils activates intracellular signaling pathways and results in a pro-inflammatory response. These same authors also utilized acutely isolated microglia from mice of various ages and demonstrated a predominance of Aβ fibril phagocytosis in young microglia (harvested from day 0 pups) in a CD47-dependent manner; however, this effect was not observed in aged microglia obtained from 6-month-old mice [35]. Thus, it has been suggested that dysfunctional CD47 may be partly responsible for the non-existent phagocytosis observed with aged microglia. Moreover, SIRP/CD47 signaling was instrumental to the cerebral infiltration of circulating monocytes. Furthermore, interactions between CD47 on endothelial cells (ECs) and SIRP expressed on monocytes promoted the transmigration of monocytes in brain endothelium, which required G1-protein activation rather than monocyte adhesion [36].

## 4. CD47, SIRP and Neurodegeneration

Neurodegeneration is the progressive loss of structure or function of neurons, which could lead to their death. New research suggests that dietary nutrition helps prevent and cure neurological symptoms in a variety of conditions [37]. Recently, interdisciplinary research in neurology and immunology has established a link between overeating and inflammation in the brain, and in particular, the hypothalamus [38]. Moreover, neurodegenerative diseases may be followed by cognitive decline and seizures that are correlated to neuroinflammation [39]. Additionally, neurodegeneration is accompanied by homeostatic changes in other CNS cells like astrocytes, microglia, and oligodendrocytes in varied forms that include cellular activation, glial scar formation, and cell death [40]. In this aspect, both CD47 and SIRP receptors have been implied in the development of neurodegenerative pathologies and processes such as neuroinflammation, multiple sclerosis, Alzheimer’s, Stroke, spinal cord injuries among others. As mentioned before, during the development of neurological disorders, CD47 has been suggested to function as a key neuroimmune regulator in responding to chronic and acute CNS injuries and its recovery [5,8]. Moreover, CD47 has broad involvement in the death of neuronal cells, neuroinflammation, and the progression of acute ischemic brain injuries [9,10]. In the following sections, we explore the importance of both CD47 and SIRP receptors in different neurodegenerative processes, and their growing importance as therapeutic targets (Table 1).

**Table 1:** CD47 effect in neurodegenerative diseases.

Neurodegenerative Diseases	CD47 Effect	Therapeutic Strategy	References
Stroke	Stimulation of disease	CD47 blocking antibody	[10,41]
Multiple Sclerosis	Function during initiation and progression has opposing effects	Modulating CD47 could be harmful and beneficial, depending on the context	[42]
Alzheimer’s Disease	Stimulation of disease	Inhibition of CD47	[43]
Spinal Cord Injury	Stimulation of disease	Inhibition of CD47	[7]
Traumatic Brain Injury	Progression of brain tissue damage and promotion of neutrophil infiltration	Inhibition of CD47	[44]
Parkinson’s Disease	Mediation of protective mechanisms	Rac1/Akt activation.	[45]

### 4.1. Stroke

It is widely accepted that cerebral ischemic injuries are correlated with various inflammatory events such as the infiltration of circulating immune cells (monocytes and neutrophils) and activation of resident cells [46]. Notably, among different types of leukocytes, neutrophils, which are the primary subtype of polymorphonuclear (PMN) leukocytes, are considered the first cells that undergo infiltration into the ischemic brain. Subsequently, the extravasated PMN leukocytes release lipid peroxidation products and reactive oxygen species and promote the disruption of the BBB, edema, vascular blockage, and infarction development [47]. Moreover, it has been suggested through in vitro experiments that the increase in the expression of adhesion moleculex may occur via CD47 signaling on the endothelium [48]. Such experimental results have advanced the premise that CD47 may possibly represent a potential anti-inflammatory target for the treatment of stroke.

Similarly, Jin et al. 2009, addressed the hypothesis of whether lack of the gene for CD47 would decrease the damage to focal ischemic brain injury [10]. Among its results, the authors determined through western blot, the absence of CD47 in the brains of the CD47 knockout mice, a significative loss of claudin-5 in the ischemic brains of the wild type mice when compared with CD47 knockout mice and an important reduction in neutrophil extravasation into the brain parenchyma in the CD47 knockout mice. Finally, a recent study in mice [41] showed that the use of CD47 blocking antibody speeded hematoma clearance and reduced brain injury after intracerebral hemorrhage (ICH), suggesting a possible clinical application in human patients. These combined results suggest the broad contribution of CD47 to neuroinflammation, as well as the involvement of integrin-associated-proteins in the promotion of neutrophil extravasation, MMP-9 up-regulation, brain swelling, and overall injury to the acute ischemic brain [10].

### 4.2. Multiple Sclerosis

Multiple sclerosis (MS) is one of the most complex neurodegenerative diseases with a heterogeneous pathology, wherein injury and repair frequently occur concurrently in the CNS tissue. Moreover, this process is frequently associated with significant inflammation in the myelinated areas of the CNS in the acute phase of the disease [49,50]. For this reason, there is a focus on a more complete elucidation of the molecular signature of MS through a rigorous analysis of genes, lipids, antibodies, and proteins involved in its development [51,52].

One investigation compared the proteomic and transcriptomic results from MS lesions with the same pathology and revealed CD47 downregulation at the messenger RNA level and lower protein levels [53]. Moreover, immuno-histochemical results from the above study demonstrated CD47 expression in foamy macrophages, reactive astrocytes in active MS lesions, and normal myelin. Han et al. [42] showed that CD47^−/−^ mice were refractory to experimentally induced autoimmune encephalomyelitis (EAE) and suggested that this was caused by the failure of immune cell activation following immunization with the myelin antigen. In contrast, using a monoclonal antibody against CD47 in the mice at the peak time of paralysis worsened EAE severity and enhanced immune activation in the peripheral immune system [42]. Additionally, in vitro assays have demonstrated that myelin phagocytosis is promoted by blocking CD47 and that this effect is dependent upon SIRPα. As a result, phagocytosis and immune regulation are considered the primary mechanism(s) for CD47 signaling involved with autoimmune-based neuroinflammation. Thus, it has been concluded that CD47 has a dual role, with discordant impacts on the pathogenesis of EAE [42].

Phagocytosis of intact myelin, or myelin debris, by macrophages, has been shown to be enhanced by the complement protein 3bi (C3bi)-receptor 3 (CR3) interaction and suppressed by the CD47-SIRPα interaction. For example, according to results by Bruck and Friedel, when C3bi becomes attached to the Fc domains of anti-myelin debris, specific antibodies opsonized the myelin debris and accelerated phagocytosis via CR3+ macrophages [54,55,56].

Different researches have suggested the importance of CD47 in MS in macrophages around the active lesions and foamy macrophages and activated astrocytes around the active MS lesions [5,6,33,57]. An initial study by Gitik et al. [33] reported that recombinant anti-CD47 antibodies opsonized the CD47+ myelin debris and accelerated F_C_γR-mediated phagocytosis by SIRPα+ macrophages, suggesting that CD47 protein expressed by either myelin debris, or intact myelin, could be one of the crucial clues associated with the molecular dynamics of CNS repair during the course of demyelination in several neurodegenerative diseases [33]. Similarly, Mahesula et al. [57], using quantitative tandem mass spectrometry-based proteomics at numerous time points, reported a correlation of expression levels of CD47 and other related proteins with importance in the disease progression in the EAE animal model of MS, including MBP:223-228, which corresponded to the basic myelin protein, and MIF:79-87, which corresponded to a pro-inflammatory cytokine that suppresses migration of macrophages [57].

Moreover, Gao et al. examined how blocking CD47 with CD47-Fc fusion protein might affect the prevention and healing of EAE via the infiltration of Th17 cells into the CNS [6]. It was found that CD47 deficiency had no direct influence on migrating Th17 cells. In addition, it was found that inhibiting the degradation of iNOS by CD47 deficiency in the proteasome of macrophages (via the activation of Src) resulted in a greater rate of NO production and the suppression in the production of inflammasome activation-induced IL-1β by iNOSl. These results based on blocking CD47 may suggest a potent treatment strategy for the control of EAE progression [6].

### 4.3. Alzheimer’s Disease

During Alzheimer´s disease (AD) development, there is a progressive accumulation of amyloid-β (Aβ) peptides that ultimately form neuronal deposits called senile plaques on the outer surfaces of neurons and cause neuronal death and glial activation [58]. It has been assumed that Aβ binding to the plasma membrane is one of the key steps in developing AD, being the formation of Aβ plaques one of the primary triggers for the degeneration of neurons [59,60,61]. Additionally, multiple published reports suggest that NO generated by endothelial cells, as well as neuronal constitutive NOS, may play a neuroprotective role in the course of Aβ-induced cell death, whereas NO produced via iNOS activation appears to play a neurotoxic role due to the inflammatory response elicited by the overproduction of reactive nitrogen species [62,63,64]. It has also been reported that the increased levels of constitutive NO formed by iNOS protects beta-amyloid transgenic mice from the progression of a majority of humanoid symptoms characteristic of AD [65,66]. Moreover, it has been demonstrated that in the case of genetic crossing through an iNOS-null background, mice exhibit widespread tau pathology related to the areas of dense microvascular amyloid deposition. In this aspect, according to the findings of previous studies, Thrombospondin 1 (TSP1) binding to CD47 and CD36, mitigates soluble guanylate cyclase (sGC) activity and cGMP concentrations resulting in NO signaling inhibition in vascular cells [67]. Interestingly, different studies have attributed the protective contribution of NO to the NO/sGC/cGMP/cGK signaling cascades as it relates to the pathogenesis of AD [68,69]. In this aspect, another study has demonstrated the interaction between Aβ and the cell surface receptors CD47 and CD36, which causes TSP1 to inhibit the activation of sGC, suggesting a protective role for CD47 against Aβ activity [70]. Importantly, both receptors, CD36 and CD47, are considered crucial for Aβ to inhibit the accumulation of cGMP. Such results reflect the induction of the CD47-dependent signal via the interaction between Aβ and CD36, which inhibits the activation of sGC. Therefore, the findings described above, when taken together with the inhibition of free fatty acid transfer via CD36, provide a molecular rationale of how Aβ could potentially be involved in the NO signaling deficiency related to AD [70].

On the other hand, some investigations have suggested the involvement of mast cells in AD. Mast cells have been observed in greater numbers in AD brains in particular, close to the amyloid deposits [71]. Because mast cells express the β_1_-integrin subunit and the CD47 receptor [43,72], phagocytosis may include a cell surface complex similar to the complex described previously for microglial cells. It is important to note that numerous mast cell mediators are secreted by activated microglia, which ultimately contributes to chronic inflammation and neurotoxicity [73]. In this aspect, Niederhoffer et al. conducted a study [43], and their results showed a decrease in Aβ1-42 and fibrillar Aβ1-40 exocytosis by pretreatment with pertussis toxin, as well as antibodies against the CD47 receptor and β_1_-integrin subunits. In summary, these authors concluded that Aβ-induced activation of mast cells functions through a CD47/β_1_-integrin membrane complex coupled with G_1_-protein. These findings support the hypothesis that mast cells, similar to microglial cells, may significantly contribute to the pathogenesis of AD [43].

Finally, Karki and Nichols [74] also reported a basic contribution of CD47 to the release of microglia cytokines triggered by soluble Ab(1–42) protofibrils. In his study, the pretreatment of primary murine microglia with the CD47 antagonist peptide 4N1K significantly inhibited interleukin-1β (IL-1β) and tumor necrosis factor-α (TNFα) secretion stimulated by Ab(1–42) protofibrils. The findings by Karki et al. demonstrated that the microglial proinflammatory response to Aβ(1–42) protofibril is not dependent on CD47 and that 4N1K demonstrates CD47-independent inhibitory activity [74]. In conclusion, the presented combined results suggest an important role for CD47 and SIRPα in Alzheimer’s development and progression.

### 4.4. Spinal Cord Injury

**Figure 3:**
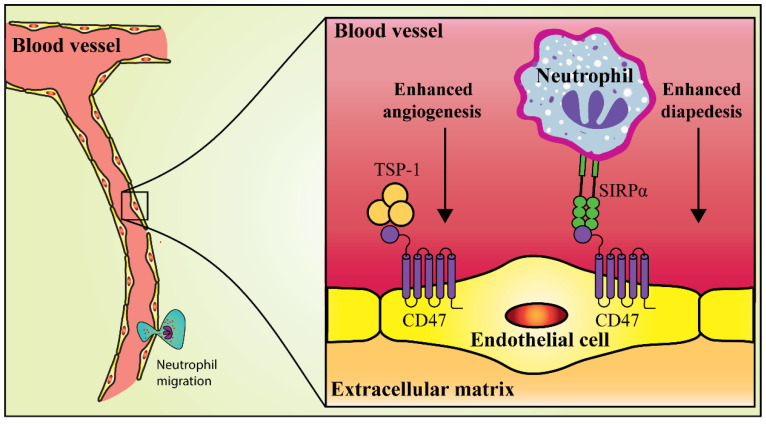
Improvement of functional recovery from contusive thoracic SCI by CD47 inhibition. CD47 suppression increases the vascular patency following the SCI. Moreover, CD47 suppression reduces neutrophil extravasation into the contusion. For this reason, CD47 has been considered a new treatment target to treat SCI. Adapted from [7].

Spinal cord injury (SCI) can induce a chronic wound state that undergoes expansion and maintains demyelination associated with an impaired recovery and progressive degeneration accompanied by maladaptive inflammation, and macrophage activation [75]. A preliminary study showed that TSP-1 activation of CD47 induced apoptosis using a caspase-dependent and -independent mechanism in cultivated cerebral cortical neurons and NB4 cells [76]. Moreover, TSP-1-mediated activation of CD47 increased cytotoxicity in CNS-derived EC cultures, although the alteration in the proliferation of the ECs was not evaluated [76]. Similarly, Myers et al. [7] demonstrated that CD47 bound to TSP-1 inhibits angiogenesis and that CD47 binding to SIRPα facilitated neutrophil diapedesis through ECs to the injured areas in a mice model. In the same study, functional improvements were not observed in TSP-1^−/−^ mice when compared to wild-type mice, although CD47^−/−^ mice exhibited greater locomotor development and sparing of white matter following contusive SCI. Moreover, the deletion of either CD47 or TSP-1 enhanced vascularity in the acute epicenter of contused mice, while the deletion of CD47 alone reduced neutrophil diapedesis and enhanced microvascular perfusion. Lastly, using an ex-vivo model of the CNS microvasculature, it was shown that CD47^−/−^ derived microvessels (MVs) significantly exhibited adherent wild-type or CD47^−/−^ neutrophils on the endothelial lumen, while the wild-type-derived MVs did not. Such a condition reflects a deficiency in diapedesis, which is mediated by the loss of CD47 expression on the ECs. Interestingly, in-vitro transmigration assays have demonstrated the contribution of SIRPα to neutrophil diapedesis via the EC monolayer [7]. These results suggest that CD47 partly improves the functional recovery from SCI by enhancing vascular patency and decreasing SIRPα-mediated neutrophil diapedesis rather than abolishing TSP-1-mediated antiangiogenic signaling (Figure 3).

More recently, Qi et al. [77] investigated the effects and probable mechanisms of miR-34a on neuron apoptosis generated by SCI. Their results showed that the injection of miR-34a agomir and/or si-CD47 could suppress neuronal cell apoptosis, with decreased levels of pro-apoptotic protein (cleaved caspase-3 and Bax) a reduced apoptotic index (AI), and an increased expression of anti-apoptotic proteins (Bcl-2 and Mcl-1). This combined body of evidence, clearly suggests the importance of CD47 and SIRPα in SCI.

### 4.5. Traumatic Brain Injury (TBI)

Some studies have shown the involvement of two crucial mechanisms in the pathologic cascade following traumatic brain injury (TBI); namely, an initial stage consisting of leukocyte-mediated BBB damage and neuroinflammation, which is followed by vascular remodeling or angiogenesis and synapse plasticity during the later stage. Together, these processes jointly determine the medical consequences (i.e., the biochemical and physiological changes) following a TBI [78,79].

Regarding CD47, a preliminary study employing microarray analysis in rats with induced TBI by head trauma demonstrated an increase in the expression of C1ql2, Cbnl, Sdc1, Bdnf, MMP9, and Cd47 genes, compared with controls, suggesting the importance of these genes during the response of the brain to TBI development [80]. Moreover, different studies have reported the crucial role of CD47 in vascular pathophysiology following brain injury, specifically in the transendothelial migration of leukocytes mediated by the SIRPα-CD47 interaction, as well as in the anti-angiogenic effects exerted via the interaction of TSP-1 with CD47 [81,82]. More recently, Zhao et al. established that CD47 mediated early neutrophil brain infiltration and late brain vascular remodeling following TBI [44]. This research used a controlled cortical impact (CCI) instrument and a CD47 knock-out mice model. Interestingly, knock-out mice exhibited significantly less brain neutrophil infiltration at 24 h, up-regulation in the level of VEGF expression in the perilesional cortex at 7 and 14 days, and enhanced blood vessel density at 21 days following the TBI when compared to these wild-type mice. Moreover, their findings revealed that CD47 knockout remarkably decreased sensorimotor function deficits and brain lesion volume at 21 days following the TBI. Therefore, these combined results suggest that CD47 has a role in the pathophysiology of TBI; specifically, in neutrophil infiltration of the brain, expansion of damage in brain tissue, injury of cerebrovascular remodeling, and recovery [44].

### 4.6. Parkinson’s Disease

Numerous published articles on the pathogenesis of Parkinson’s disease (PD) suggest a contribution of T-lymphocytes to the neuroimmune activity in the brain of PD patients [83,84,85]. Other studies showed the infiltration of T-lymphocytes into the brain and locations surrounding the degenerating dopaminergic neurons with activated microglia in PD patients, as well as in experimental models of PD [86,87,88]. For example, a study by Huang et al. [45] showed the ability of Treg cells (regulatory T cells) to directly protect dopaminergic neurons against MPP^+^ (1-methyl-4-phenylpyridinium) treatment as well as inflammatory responses by glial cells, through the interaction between the transmembrane proteins SIRPα and CD47 [45]. In this study, it was also found that SIRPα knockdown in VM neurons resulted in a decrease in neuroprotection by Treg cells. It was also established that Treg cells and VM neurons activated the Rac1/Akt signaling pathway in the VM neurons. Consequently, suppression of Rac1/Akt signaling in VM neurons compromised Treg cell neuroprotection. Finally, the overall conclusion from the study was that Treg cells protect dopaminergic neurons against MPP^+^ neurotoxicity by a cell-to-cell contact mechanism underlying the CD47-SIRPα interaction and Rac1/Akt activation [45].

In addition, transwell co-cultures of VM neurons and Treg cells were utilized to assess the effects of the Treg cytokines TGF-β1 and IL-10 on dopaminergic neurons. According to their ‘live-cell’ imaging experiments, there was dynamic contact of Treg cells with VM neurons that were stained with CD47 and SIRPα respectively. Following the silencing of either CD47 in Treg cells or the silencing of SIRPα in VM neurons, these same authors investigated dopaminergic neuronal loss using tyrosine hydroxylase (TH)-immuno-reactive cells. The results of these experiments confirmed that Treg cells prevented MPP^+^-induced dopaminergic neuronal loss, as well as inflammatory responses by glial cells [45]. However, TGF-β1 and IL-10 secreted from Treg cells did not significantly prevent MPP^+^-induced dopaminergic neuronal loss in the transwell co-cultures of VM neurons and Treg cells. Furthermore, CD47 and SIRPα were expressed by Treg cells and VM neurons, respectively. It is very important to note that Huang et al. [46] demonstrated that silencing the CD47 gene in Treg cells impaired the ability of the cells to protect dopaminergic neurons against MPP^+^ toxicity. Similarly, SIRPα knockdown in VM neurons resulted in a decrease in neuroprotection by Treg cells. These same authors also demonstrated that the CD47-SIRPα interaction between Treg cells and VM activated the Rac1/Akt signaling pathway in VM neurons. These combined results suggest that the CD47-SIRPα interaction and Rac1/Akt activation appear as a promising mechanism in the treatment of PD.

## 5. Conclusions and Future Directions

A growing body of evidence has shown the importance of CD47/SIRPα interaction in the brain, both in normal and pathological conditions, specifically for neutrophil infiltration of the brain, expansion of damage in brain tissue, injury of cerebrovascular remodeling, and recovery. Numerous studies have reported increased expression of the CD47 receptor in a variety of neurological diseases. In the present review, we have highlighted the current evidence regarding the importance of CD47/ SIRPα in pathologies like stroke, Multiple Sclerosis, Alzheimer’s, spinal cord injuries, TBI, and Parkinson’s. Is possible that additional brain pathologies could be influenced through CD47/ SIRPα related processes. In this aspect, a study by Ohnishi et al. [89] using transgenic mice with a truncated form SIRPα that lacked most of its cytoplasmic region, manifested prolonged immobility in the forced swim (FS) test suggesting an association of this receptor in depression-like behavior. However, a major challenge in this aspect would be to properly translate the results established in animal models to human clinical studies.

The differential effects of CD47 on peripheral immune cells and its impact on the CNS have created numerous intriguing issues regarding its exact biological functions. Additional studies are needed in order to understand the precise functions of CD47/SIRPα in astrocytes, neurons, and myelin. For example, a recent study in human and mice glioblastoma cell lines showed that the combined CD47 blockade with temozolomide resulted in a pro-phagocytosis effect against tumorigenic cells [90] compared with normal astrocytes. Consequently, further research pertaining to the specific functions and roles of CD47 and SIRP is required prior to its exploitation as a potential target in the treatment of various neurodegenerative diseases and cancer.

## Abbreviations

SHPS-1	SH2-domain bearing protein tyrosine phosphatase substrate-1
ALL	Acute lymphoblastic leukemia
NHL	Non-Hodgkin’s lymphoma
MM	Multiple myeloma
AML	Acute myeloid leukemia
IAP	Integrin associated protein
SIRP	Signal regulatory protein
NIRegs	Neuroimmune Regulatory Proteins
TNF	Tumor necrosis factor
FAS	*F*S-7-associated surface antigen
ITIM	Immuno-receptor tyrosine-based inhibition motif
SHP	SH2 domain-containing phosphatase
ECs	Endothelial cells
PMN	Polymorphonuclear
ICH	Intracerebral hemorrhage
MS	Multiple sclerosis
EAE	Experimentally-induced autoimmune encephalomyelitis
C3bi	Complement protein 3bi
AD	Alzheimer´s disease
Aβ	Amyloid-β
TSP1	Thrombospondin 1
sGC	Soluble guanylate cyclase
IL-1β	Interleukin-1β
TNFα	Tumor necrosis factor-α
SCI	Spinal cord injury
MVs	Microvessels
AI	Apoptotic index
TBI	Traumatic Brain Injury
CCI	Controlled cortical impact
PD	Parkinson disease
Treg cells	Regulatory T cells
MPP^+^	1-methyl-4-phenylpyridinium
TH	Tyrosine hydroxylase
FS test	Forced swim test
SHIP	SH2 domain-containing inositol phosphatase
